# The governmental health policy-development process for Syrian refugees: an embedded qualitative case studies in Lebanon and Ontario

**DOI:** 10.1186/s13031-019-0231-z

**Published:** 2019-10-21

**Authors:** Ahmad Firas Khalid, John N. Lavis, Fadi El-Jardali, Meredith Vanstone

**Affiliations:** 1 Health Policy PhD Program, McMaster University, Hamilton, ON Canada; 2 Department of Health Research Methods, Evidence and Impact, McMaster University, Hamilton, ON Canada; 3 Centre for Health Economics and Policy Analysis, McMaster University, Hamilton, ON Canada; 4 McMaster Health Forum, McMaster University, Hamilton, ON Canada; 5 Department of Political Science, McMaster University, Hamilton, ON Canada; 6 Department of Health Management & Policy, American University of Beirut, Beirut, Lebanon; 7 Center for Systematic Review in Health Policy and Systems Research (SPARK), American University of Beirut, Beirut, Lebanon; 8 Knowledge to Policy (K2P) Center, American University of Beirut, Beirut, Lebanon; 9 Department of Family Medicine, McMaster University, Hamilton, ON Canada

**Keywords:** Health policy, Syrian refugees, Case study, Lebanon, Ontario

## Abstract

**Background:**

The unprecedented amount of resources dedicated to humanitarian aid has led many stakeholders to demand the use of reliable evidence in humanitarian aid decisions to ensure that desired impacts are achieved at acceptable costs. However, little is known about the factors that influence the use of research evidence in the policy development in humanitarian crises. We examined how research evidence was used to inform two humanitarian policies made in response to the Syrian refugee crisis.

**Methods:**

We identified two policies as rich potential case studies to examine the use of evidence in humanitarian aid policy decision-making: Lebanon’s 2016 Health Response Strategy and Ontario’s 2016 Phase 2: Health System Action Plan, Syrian Refugees. To study each, we used an embedded qualitative case study methodology and recruited senior decision-makers, policy advisors, and healthcare providers who were involved with the development of each policy. We reviewed publicly available documents and media articles that spoke to the factors that influence the process. We used the analytic technique of explanation building to understand the factors that influence the use of research evidence in the policy-development process in crisis zones.

**Results:**

We interviewed eight informants working in government and six in international agencies in Lebanon, and two informants working in healthcare provider organizations and two in non-governmental organizations in Ontario, for a total of 18 key informants. Based on our interviews and documentary analysis, we identified that there was limited use of research evidence and that four broad categories of factors helped to explain the policy-development process for Syrian refugees – development of health policies without significant chance for derailment from other government bodies (Lebanon) or opposition parties (Ontario) (i.e., facing no veto points), government’s engagement with key societal actors to inform the policy-development process, the values underpinning the process, and external factors significantly influencing the policy-development process.

**Conclusions:**

This study suggests that use of research evidence in the policy-development process for Syrian refugees was subordinate to key political factors, resulting in limited influence of research evidence in the development of both the Lebanese and Ontarian policy.

## Background

Globally, there has been an unprecedented amount of resources dedicated to humanitarian aid [1]. During a crisis, humanitarian aid often includes the provision of health services, protection, shelter, and food. Using evidence to develop humanitarian aid policy can increase the effectiveness and efficiency of interventions [2–4]. However, little is known about the factors that influence the use of research evidence on policy development in humanitarian crisis. A large investment has been made towards ensuring that humanitarian aid is adequately responding to crises and, thus, a deeper understanding of the factors that influence the use of research evidence in the policy-development process is required in order to ensure that this investment is maximized. This deeper understanding could help tailor future policy-development processes so that they may achieve their intended results. We examined how two policy cases were made in response to the Syrian refugee crisis in Lebanon and Ontario.

The Syrian conflict started in the spring of 2011 as a result of a civil war and has caused an estimated 6.6 million people to be displaced within Syria and over 5.6 million refugees seeking safety in Turkey, Lebanon, Jordan, Canada, and beyond. This large exodus of people has placed a strain on host countries’ health systems [5]. The most prevalent medical problems, which Syrian refugees face, include trauma related mental health disorders, skin, digestive system, and respiratory diseases [6]. In addition, many Syrians have chronic health conditions. For instance, 50% of Syrian refugee households in Lebanon report at least one member living with a non-communicable disease (NCD) [7]. The management of NCDs requires a long-term approach with often costly and complex solutions. Along with managing NCDs, the Syrian refugee crisis presents a complex set of issues for policymakers to consider, including dealing with mass causalities and injuries and with infectious-diseases outbreaks [8]. This makes it imperative that we use the best available research evidence to inform policy decisions so that money, time, and resources are invested in effective solutions [7, 9, 10]. In addition, evidence use in health policy-making ensures that the use of evidence reaches back to populations of concerns in humanitarian settings [11].

What makes decision-making in humanitarian settings unique is the high levels of stress, often in intense and sometimes dangerous situations. Research evidence can help decision-makers respond in a timely manner in such situations. However, the use of research evidence to help respond to crises is not always straightforward. A culture built on immediate action with a traditionally heavy reliance on professional judgement may not be conducive to using evidence to inform decision-making in crisis zones [12]. For example, when faced with an unexpected event, decision-makers may draw on their personal experiences to inform their decisions, partly because of a perceived gap in the evidence base on humanitarian action [13–15]. In addition, the humanitarian domain can be conflicted on what constitutes evidence because the dividing lines among operational data, theory, and evidence are perceived as unclear. Humanitarian aid organizations may primarily rely on data stemming from their ground operations instead of considering the data alongside existing research evidence.

Policymaking is a highly complex process that requires multiple inputs (e.g., research evidence, common sense knowledge) and can be dependent on the social, political, and historical context in which it occurs [16–25]. An area rarely studied in the health policy literature is how research evidence is used in policy development in crisis zones. We define research evidence as the output of research that has been conducted in a systematic way and reported in a transparent manner. Generally speaking, research evidence can inform policymaking in three ways: instrumental, conceptual, and symbolic [26, 27]. These concepts can be applied to policymaking in the Syrian refugee crisis. For example, policymakers may instrumentally use effectiveness and cost-effectiveness studies to decide which drug is best to treat diabetes among the Syrian refugee population. Additionally, policymakers may generally use an overview of reviews of humanitarian-aid interventions to help them to identify broad areas where they may need to give greater or lesser attention. Finally, policymakers may symbolically use evidence when announcing that they will allow nurses in Syrian refugee camps to prescribe diabetes medication because of a shortage of primary-care physicians and only later look to see whether there is research evidence to suggest that nurses can safely and effectively prescribe diabetes medication [26, 27].

This study focuses on examining the factors that influence the use of research evidence in the governmental health policy-development processes in Lebanon and Ontario for two main reasons. First, the role of research evidence in policymaking is often limited [17]. Even when research evidence is used by policymakers, such evidence use is often affected by political processes. Studies suggest that policymakers tend to rely on common sense and personal experiences, and that they are concerned with recognition and re-election [22, 24, 25]. Second, the real-life context in which policymakers are developing policies to respond to a crisis has rarely been in the literature [28]. For these two reasons, particular attention needs to paid to how evidence is used in policy development around humanitarian crises; the case studies of Lebanon and Ontario will illustrate how this played out in two different contexts.

## Methods

### Study design

We used a qualitative embedded case study design. The context of this case study is the host countries response to the Syrian refugee crisis. The case study is bounded by the following timeframe: the 2011 civil war outbreak in Syria, which prompted the refugee crisis, and the release of the key policies in October 2016. In Lebanon, the refugee crisis overlaps with the 2011 conflict in Syria and is still ongoing; however, in Ontario, it was primarily perceived as a crisis in 2015 when the decision was made to accept Syrian refugees. The case studied is the factors that influenced the use of research evidence in health policy-development processes to prepare health systems to respond to an influx of Syrian refugees. The embedded cases consist of recent key policies that host countries have developed to deal with the health of Syrian refugees. Both policies represent the most significant strategy dealing with the health system’s response made in the host country in the last 4 years.

### Embedded cases 

We included Lebanon and Canada’s province of Ontario because they have been among the top jurisdictions taking in registered Syrian refugees since the beginning of the crisis. This ensured that we are examining a country where the crisis has had a significant impact. Additionally, both countries have in place explicit mechanisms to support the use of research evidence in policymaking (e.g., Knowledge to Policy (K2P) center in Lebanon and McMaster Health Forum in Ontario), and where access to the key individuals and the documents required to conduct a robust case study could be facilitated through our contacts involved with these explicit mechanisms.

Our first embedded case is Lebanon’s 2016 Health Response Strategy (HRS). In response to the influx of Syrian refugees in Lebanon and to address the pressures placed on the Lebanese health system, the Ministry of Public Health (MoPH) made the decision to develop a Health Response Strategy (HRS), which was released in 2015 and subsequently updated in October 2016. It served two interdependent strategic objectives: 1) to harness primary, secondary, and tertiary care to address the essential health needs of the displaced Syrians and host community; and 2) to strengthen national institutions and capacities and thereby enhance the resilience of the health system.

The second embedded case is Ontario’s Ministry of Health and Long -Term Care (MOHLTC) 2016 Phase 2: Health System Action Plan, Syrian Refugees, which included a set of policies to prepare the province of Ontario for managing the current and future health status of Syrian refugees who moved to the province. It provided guidance on: roles and responsibilities of specific health system partners, general guidance and considerations for Syrian refugee healthcare, and resources available to support continuing Syrian refugee healthcare delivery.

### Data sources 

We identified and recruited key informants based on their involvement in the development of both embedded cases. These key informants included: 1) senior decision-makers, staff employed by Lebanon’s and Ontario’s Ministries of Health, international agencies (e.g., UN system organizations), and at non-governmental organizations (e.g., Red Cross); 2) policy advisors who helped inform the health policy-development process; and 3) healthcare providers who were involved with the development of the policies. The second stage involved snowball sampling by which research participants in the first stage were asked to identify any additional informants. We reviewed the policy documents to obtain the names of these key informants and communicated with key individuals familiar with the policy-development process to ensure that we found the most appropriate individuals to interview. Given the limited pool of potential participants with knowledge of the policy-development process, we aimed to complete 10 interviews for each country. A total of 18 informants were willing to participate in the interviews across the two settings (Table 1).

**Table 1:** Characteristics of key informants interviewed to understand the policy-development process

Policy	% (n)	Key informant position	Organizational affiliations	Organizational types
Health Response Strategy	78% *(n* = 14)	Senior decision-maker (*n* = 13) Policy advisor (*n* = 1)	Ministry of Public Health United Nations United Nations High Commissioner for Refugees United Nations International Children’s Emergency Fund World Health Organization	Government agency (*n* = 8) International agencies (*n* = 6)
Phase 2 Ontario Health System Action Plan: Syrian Refugees	22% (*n* = 4)	Healthcare provider (*n* = 2) Senior decision-maker (*n* = 2)	Canadian Centre for Refugee & Immigrant HealthCare Canadian Red Cross Crossroads Clinic for Refugees	Healthcare provider (*n* = 2) NGO (*n* = 2)

In addition to interviewing key informants, we also reviewed publicly available documents and media articles that spoke to the factors that influence the health policy-development process under study. The type of documents included, but were not limited to: governments’ and intergovernmental organizations’ annual reports and related policy documents (i.e., United Nations High Commissioner for Refugees health access and utilization survey among Syrian refugees in Lebanon), media articles using LexisNexis, transcripts of legislative debates (e.g., Hansard in Ontario), and published literature using PubMed. Google was searched for other document types (e.g., memos, briefs, etc.,) as was the Internet Archive for documents that are no longer available on internet websites (an additional file shows this in more detail (see Additional file 1)).

### Data collection methods

We used a semi-structured interview guide that included a number of open-ended questions, allowing the participant to direct the initial content and flow of the interview (an additional file shows this in more detail (see Additional file 2)). The 3-I framework, a political science framework with its three categories of influences on the policy-making process — ideas, interests, and institutions — was used as a guide to elicit responses around the political factors that influenced the health policy-development process. Participants were given the option of phone or in-person interviews. Interviews typically lasted 30–45 min. Each was recorded and transcribed verbatim and the written transcriptions along with any memos taken throughout the study were used for data analysis. The language of the interviews was in English, which is a language used by all of our participants.

For data collection related to the published literature and policy documents on the policy-development process for Syrian refugees, a search strategy was developed that incorporated key terms identified in the preliminary analysis of documents and archival records to develop appropriate electronic search strategies. The search was conducted in both English and Arabic languages.

### Data analysis

**Fig. 1:**
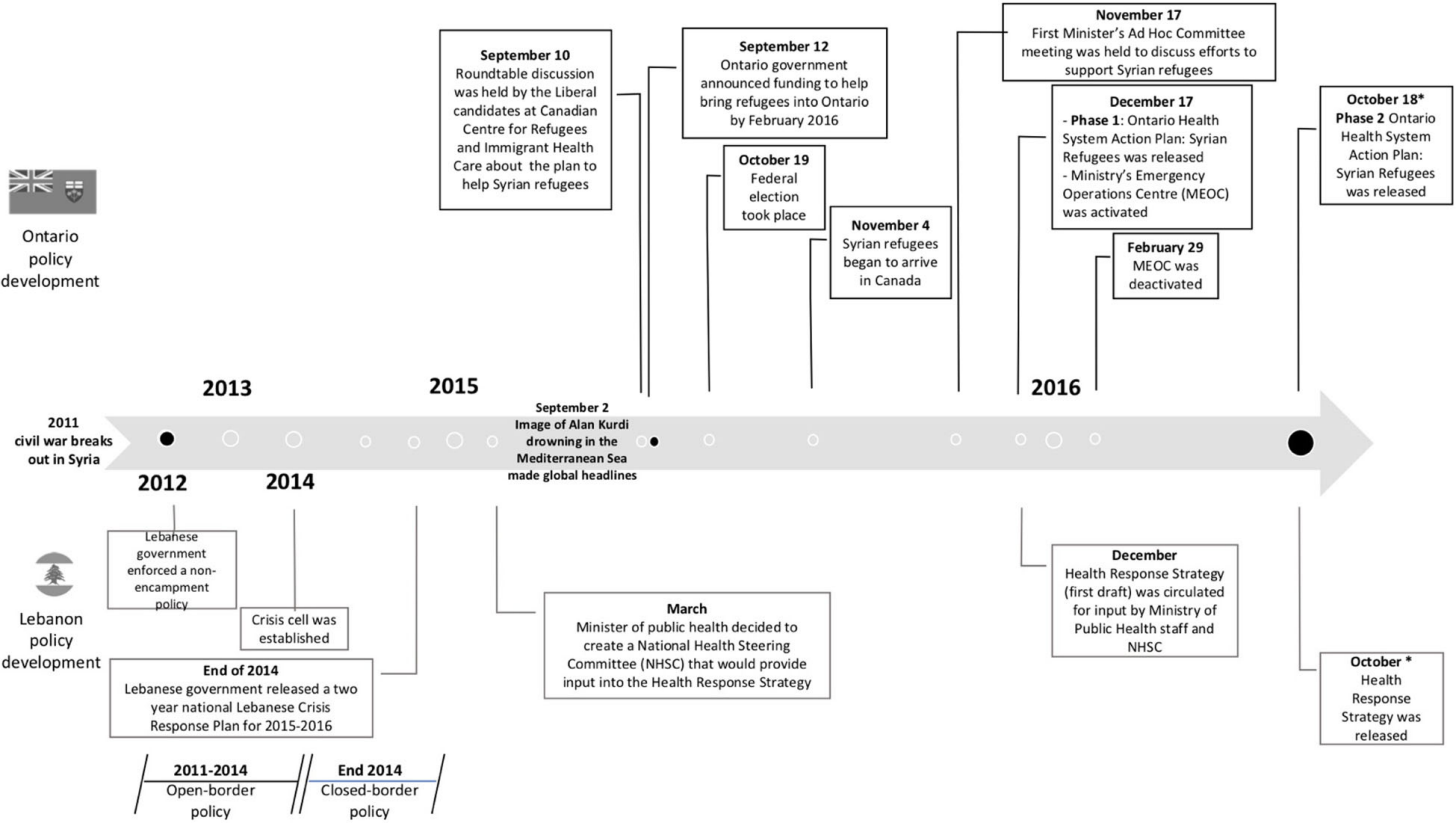
Timeline of the principal events in the policy-development process for Syrian refugees

The analytic technique of explanation building, which is a type of pattern matching, was used with the goal of using the case study analyses to build an explanation about the case [29]. It is similar to causal mechanisms in political science, which helps understand under what conditions these two policies were made. Using an existing political science theoretical framework, the 3-I framework with its three categories of influences on the policy-making process — ideas, interests, and institutions [30], helped to explain the policy-development process [16]. The documentary analysis was conducted to help us develop a timeline of the principal events in the policy-development process (Fig. 1), and to fill in any gaps in our understanding of how the policy-development process unfolded.

## Results

Our results section starts with a brief description of the key informants we interviewed across the two settings to arrive at a comprehensive understanding of the how the policy-development process unfolded. We then describe the principal events that occurred, based on our document review and information provided by our key informants. Our last two sections focus on answering our research objective of examining the factors that influence the use of research evidence in the governmental health policy-development processes for Syrian refugees in Lebanon and Ontario.

### Key informants’ profiles

Eighteen interviews were completed to understand the policy-development process for Syrian refugees in Lebanon and Ontario (Table 1). For Lebanon, we interviewed all 14 key participants identified by the MoPH as the key individuals involved in the policy-development process. For Ontario, we interviewed all three main refugee health experts identified by the MOHLTC as the only outside-of-government informants involved in the policy-development process. Our fourth informant was identified through snowball sampling. We were unable to interview anyone within the MOHLTC because they declined our request for interview. In light of this decline, we reached out to a contact working at the Local Health Integration Networks (LHINs), who informed us that given the short timeline, the policy was developed entirely within the MOHLTC with consultation from the three external experts we interviewed.

### Timeline of principal events

Four key observations can be drawn from the timeline of the principal events in the policy-development process for Syrian refugees (Fig. 1). First, Syrian refugees began to arrive in Ontario on November 4, 2015, while in Lebanon Syrian refugees started arriving when the civil war broke out in Syria in 2011. Second, Ontario accepted a total of 10,210 Syrian refugees while Lebanon had over a million registered Syrian refugees and likely many more unregistered refugees at the time of the policy-development process [31, 32]. Third, both jurisdictions solicited advice from various societal key players (e.g., NGOs, UN agencies, etc.), through roundtable discussions (Ontario) and the creation of the National Health Steering Committee (Lebanon). Finally, both jurisdictions released the first draft of their respective health policies on December of 2015 and both released the updated version of the policies in October 2016.

Our document analysis indicated that in Lebanon, there were three key knowledge production and translation efforts by the Knowledge to Policy (K2P) Center and by the Center for Systematic Reviews on Health Policy and Systems Research in 2014. First, a priority-setting exercise resulted in the production of systematic reviews to address the health needs of Syrian refugees [33, 34]. Second, the production of a briefing note allowed for the contextualizing of global research evidence applied to the Lebanese health system [4]. Finally, a policy dialogue arranged and organized by the K2P center and supported by the Ministry of Public Health in Lebanon helped bridge the views, experiences and tacit knowledge of key stakeholders with research evidence [18]. Based on the briefing note and policy dialogue, the Lebanese MoPH recruited a refugee health coordinator and a National Health Steering committee to develop the Health Response Strategy. None of these efforts were identified explicitly during our interviews by our Lebanese key informants.

### Research evidence influencing the policy-development process

There was limited use of research evidence to inform the policy-development process in Lebanon and Ontario. In Lebanon, key informants discussed the scarcity of available research evidence to inform the policy-development process. A senior decision-maker working at MoPH stated:

*“there were clear gaps in available research evidence. When we found a gap in the evidence we did not commission a study because of concern of funds going to completing studies instead of services when services were severely underfunded. In the context of this emergency, we had to get the data ourselves without having flawless research paper with a perfect methodological method”*

In Ontario, a healthcare provider discussed the scarcity of available research evidence and highlighted that research evidence was mainly used to clarify the health issues of Syrian refugees and not necessarily to inform the policy options and implementation considerations stating:

*“We had no available literature on what the health conditions of Syrian refugees were. What was really helpful for us on the ground was having a better understanding of the epidemiological conditions in those Syrian refugee populations in Turkey, Lebanon, and Jordan. That required us to look at whatever available evidence we had at the time which included some cross-sectional disease prevalence studies and make policy decisions based on that”*

#### Information, other than research, informing policymaking

A common finding across our two settings was that both governments drew on inputs from a variety of sources. The scarcity of research evidence led the Lebanese MoPH to rely primarily on data obtained from two main sources: MoPH databases (e.g., Maternal Neonatal Mortality Notification System) and reports from other organizations (e.g., UN Vulnerability Assessment of Syrian Refugees (VASyR)). The data were used in specific and direct ways to learn about the health needs of, and how to support, Syrian refugees (i.e., instrumental use of evidence). This highlights the importance for up-to-date and accessible databases during a crisis. A senior decision-maker commented on the use of data by the MoPH to inform the policy-development process stating:

*“the MoPH looked at what data was available in their hospital observatory system which was providing some quite reliable data and they had some data from network of primary healthcare and then there was other data from other studies being done. For example, there was the VASyR survey led by UNCHR and others that came up with interesting data on health aspects (e.g., access to healthcare and disease profile). There was the Hopkins survey looking at access and disease profile among refugees and host communities”*

A senior decision-maker highlighted how other sources of information (e.g., tacit and experiential knowledge) were used by the Ontario government to inform the policy-development process stating:

*“the information provided is not necessarily evidence-based reports. It is the expertise and knowledge of the wonderful staff and volunteers that we are constantly engaged with and interact with and that is the advantage of being a global organization that we can reach out to someone that is an Arabic speaking person who can help us better understand the context”*

#### Barriers in the use of research evidence in policymaking

There was a degree of consensus across the two settings that some of the main barriers that resulted in the limited use of research evidence included: short time-frame to develop the policies, and accessibility to and availability of relevant systematic reviews. These practical constraints were reported by a senior decision-maker in Lebanon stating:

*“in a crisis situation, you do not have the time to search for systematic reviews. Systematic reviews were not always available. The approach to commission systematic reviews was costly and timely. This is why data, experience and practice of key stakeholders was more important*”

Overall, our results identified that research evidence was not the main determining factor influencing the policy-development process for Syrian refugees in Lebanon and Ontario.

### Factors influencing the policy-development process

Table 2 provides a comprehensive list of the full range of likely political factors that influenced the policy-development process. The four bolded bullet points represent the most salient factors influencing the policy-development process: institutions encompassing both government structures and policy networks, ideas encompassing values about ‘what ought to be’, and external factors. We elaborate further on those four points with supporting statements from our key informant interviews.

**Table 2:** Summary of factors playing a role in policy-development process for Syrian refugees

Factor		Policy: Lebanon’s Health Response Strategy (2016)	Policy: Phase 2 of Ontario’s Health System Action Plan (2016)
Policy development	Institutions	Government structures • The Health Response Strategy fell under the sole jurisdiction of the MoPH because of is technical nature (e.g., analysis of health needs, MoPH guidelines for health institutions, budget allocations), which meant that it effectively faced no opposition in decisions about supporting Syrian refugees Policy networks • MoPH convened a National Health Steering Committee, that comprised of major international and local partners, to inform the policy-development process Policy legacies • Past Palestinian refugee camp policies resulted in an interpretive effect among the Lebanese where by camps were perceived as sources of insecurity, radicalisation and armed groups, and as places to be avoided. In order to avoid these problems, Syrian refugees have been integrated into communities; however, this has placed a strain on the health system, necessitating this strategy to help address their health needs	Government structures • Ontario’s first-past-the-post system meant that a majority Liberal government, elected in 2014, was able to act on the federal Liberal government priorities of accepting Syrian refugees without significant chance for derailment from opposition parties (i.e., it faced no veto points) Policy networks • Liberal government convened round-table discussions with key societal actors (e.g., Canadian Red Cross, Canadian Centre for Refugee and Immigrant Healthcare, and Crossroads Clinic for Refugees) to inform the policy-development process Policy legacies • Past restrictive Federal government Conservative Party immigration policies resulted in an interpretive effect among Ontarians that a more balanced immigration policy was needed for vulnerable refugees in need of assistance, especially given Canada is comprised of many immigrants who immigrated to Canada in search of a better life
	Interests	Societal interest groups • Some actors drew on their practical experiences in Syria and in Lebanon to lobby government about their preferred approaches to addressing the health needs of Syrian refugees	Societal interest groups • Some actors drew on their practical experiences in Syria and in Canada to lobby government about their preferred approaches to addressing the health needs of Syrian refugees
	Ideas	Values about ‘what ought to be’ • Lebanese values of providing safety for displaced people underpinned the policy-development process Knowledge/beliefs about ‘what is’ The government drew on inputs from a variety of sources, many of which were informed by research evidence and other types of information, such as • Lessons were drawn about how to prevent and manage future infectious disease outbreaks among Syrian refugees from prior management of measles, hepatitis A, and Leishmaniasis disease outbreaks in 2015 • Data was obtained from two main sources: first, MoPH databases (e.g., Maternal Neonatal Mortality Notification System) that included data on service utilization, human resources, immunization coverage, and disease prevalence related to displaced Syrians. Second, reports from other organizations (e.g., Johns Hopkins’ Syrian refugee’s health access survey (2015), UN Vulnerability Assessment of Syrian Refugees, and World Bank assessments) about the health needs of Syrian refugees (e.g., access to PHC services, etc.) • Analysis of NGOs’ funding initiatives demonstrated that 45% of donor funds were spent on organizational overhead costs, prompting the policy-development process to appropriately align funds and human resources and reduce overhead costs • Information from the Lebanon crisis response plan (2015–2016) helped inform sections of the Health Response Strategy (e.g., Primary healthcare (PHC) budget that included funds allocated to support mental health needs of Syrian refugees, etc.) • Tacit and experiential knowledge obtained from addressing health needs of the Palestinian refugees	Values about ‘what ought to be’ • Ontarians values such as inclusion and fairness underpinned the policy-development process Knowledge/beliefs about ‘what is’ The Ontario government drew on inputs from a variety of sources, many of which were informed by research evidence and other types of information, such as • Lessons were drawn about how to manage the health needs of Syrian refugees ‘within routine practices’ from the implementation of Phase 1 Ontario’s Health System Action Plan (2015) that primarily focused on addressing the health needs of Syrian refugees upon arrival in Ontario (e.g., primary-care provision at Toronto Pearson International Airport, which acted as the point-of-entry for refugees) • Existing research evidence were drawn that included cross-sectional disease prevalence studies among Syrian refugees in other contexts (e.g., Jordan, Lebanon, Turkey), Canadian-adapted Sphere emergency social-services guidelines about shelter and about transportation to healthcare facilities, and medical guidelines from ‘on the ground’ organizations (e.g., at Médecins Sans Frontières, United Nations High Commissioner for Refugees) • Tacit and experiential knowledge obtained through direct contact with field personnel in Jordan and Syria was used in specific and direct ways to learn about health needs and how best to provide linguistic services (e.g., Arabic interpretations) and culturally appropriate services (e.g., dietary needs) • Data about the humanitarian response plans inside of Syria was obtained from reports from other organizations (e.g., International Organization for Migration) • Insights on culturally appropriate ways of providing healthcare was obtained from conversations with newly arrived Syrian refugees
	External factors	International donors • International donors (e.g., the European Union) targeted priorities (e.g., maternal & child health) influenced the focus of the strategy. The policy-development process was intended to increase and align donor funds to specific health outcomes	Media coverage • Photo of Alan Kurdi, a 3-year-old Syrian boy who drowned on 2 September 2015 in the Mediterranean Sea when his family was escaping Syria into Europe, became a focusing event among Ontarians and the governing Ontario Liberal party that accelerated the policy-development process

#### Institutions: government structures

In Lebanon, the Health Response Strategy effectively faced no opposition because it fell under the sole jurisdiction of the MoPH National Health Steering committee: a technical committee headed by the MoPH General Director and comprised of major international and local partners and focused on informing the policy-development process (e.g., analysing health needs of Syrian refugees, reviewing MoPH guidelines for health institutions, and budget allocations). Senior decision-makers commented:

*“this strategy is produced by the MoPH with the endorsements of all stakeholders but not from higher authority. This is in comparison to Lebanon Crisis Response Plan (LCRP) where every single chapter in that plan had to get approval from every named ministry, in particular ministry of social affairs who were mandated to coordinate between all ministries to develop the plan. This leads to a lot of political factors playing a role in hindering the policy-development process”*

Similarly, Ontario’s first-past-the-post electoral system meant that a majority Liberal government, elected in 2014, was able to act on the Liberal Federal government priorities of accepting Syrian refugees without significant chance for derailment from opposition parties (i.e., it faced no veto points). A senior decision-maker highlighted the Liberal Federal government decisions about welcoming Syrian refugees by stating:

*“The fact that the* [Federal] *government was favorable got the ball rolling in a very significant way. When the Trudeau government was elected, the policy of the Liberal government, which was to open doors and receive people in need, meant that for us (i.e., senior decision-makers in Ontario) we can start the dialogue about how to best help the federal government get there”*

It is significant to highlight that the Ontario policy was supported by federally legislated funding attached to demarcated priorities for refugee health and the Lebanese policy was a bureaucratic exercise without elected official oversight. This meant that gathering support for the policies was not particularly challenging.

#### Institutions: policy networks

Second, both jurisdictions relied on policy networks to inform the policy-development process. In Lebanon, the MoPH convened the National Health Steering committee for the purpose of informing the policy-development process. A senior decision-maker at the MoPH described the role of the National Health Steering committee stating:

*“the National Health Steering committee was designed to get all the buy-ins, everyone on board, to make sure we did not miss on anything in the field. The point of the committee was to get all the perspectives on the table. It was mostly made up of the EU delegation, the World Bank, the big UN agencies all at the same table. The point was to have an open and honest conversation about the whole process, how to get money, and where it should go”*

In Ontario, the Liberal government convened round-table discussions with key societal actors (e.g., Canadian Red Cross, Canadian Centre for Refugee and Immigrant Healthcare, and Crossroads Clinic for Refugees). These round-table discussions played a crucial role in the policy-development process as stated by a senior decision-maker:

*“one of the biggest takeaways was having key stakeholders from all different areas (not just the Ministry) but the key external stakeholders like Canadian Centre for Refugee and Immigrant Healthcare, Crossroads Clinic for Refugees, Canadian Red Cross (CRC) present at the same table was very important and very influential because what that brought to the entire planning exercise was the fact that because CRC was so involved in the community sector we were able to create those linkages”*

For Lebanon and Ontario, having direct contact and interactions with key stakeholders from various organizational affiliations played an instrumental role in the policy-development process.

#### Ideas: values about ‘what ought to be’

The Lebanese values of providing safety for displaced people underpinned the Health Response Strategy with one senior decision-maker stating:

*“what propelled the policy-development process was the safety of the vulnerable people”*

Similarly, the Syrian refugee crisis spoke to Ontarians values, such as inclusion and fairness, which underpinned the policy-development process. A healthcare provider, involved in informing the policy-development process, reaffirmed this by stating:

*“this* [Ontario’s Health System Action Plan] *was made for a host of reasons, one of which to restore the soul of the nation. A nation defined where everybody has recently been touched by the refugee and immigrant experience, where by definition everybody is from somewhere else”*

A senior decision-maker highlighted the willingness of Ontarians to help displaced Syrians by stating:

*“everybody had the best intent of ensuring that the refugees are welcomed and received and healthy and are able to be supported in the best possible way”*

#### External factors

In Lebanon, international donors played an important role in influencing the policy-development process. Given the protracted nature of the Syrian refugee crisis in Lebanon, the policy-development process was seen as a way to mobilize increased funding from international donors (e.g., the European Union), and to align funding to targeted priorities (e.g., maternal and child health). A senior decision-maker highlighted the influence of international donors on the policy-development process, stating:

***“*** *the National Health Steering committee, included major donors particularly the European Union, that was very engaged. The strategy worked both ways: mobilize more funding from international donors but also some of the donors committed to certain projects, like maternal and child health, which ended up shaping the strategy”*

Our document analysis indicated that media coverage played a significant role in the policy-development process for Syrian refugees in Ontario. The photo of Alan Kurdi, a 3-year-old Syrian boy who drowned on 2 September 2015 in the Mediterranean Sea when his family was escaping Syria into Europe, became a focusing event among Ontarians and the governing Ontario Liberal party that accelerated the policy-development process [35, 36].

## Discussion

Our study provided a deeper understanding of the factors that influence the policy-development process in crisis zones and the role of research evidence in the process. This study identified four broad factors that help to explain the overall policy-development for Syrian refugees in Lebanon and Ontario: development of health policies without significant chance for derailment from other government bodies (Lebanon) or opposition parties (Ontario) (i.e., facing no veto points), government’s engagement with key societal actors to inform the policy-development process, the embedded values underpinning the process, and external factors significantly influencing the policy-development process. These different factors provide insight into the influence of political factors in policy processes in crisis zones, which could help inform future policy-development processes.

Our study found that policymakers in Lebanon and Ontario voiced similar challenges with navigating the gaps in available research evidence to inform the policy options and implementation considerations relevant to the policy-development process (e.g., reviews about strategies that should be considered in order to facilitate the necessary system changes). Policymakers raised the issue of inadequate access to systematic reviews in a short-time frame. It is a surprising finding that although evidence websites are available to support this particular issue, they were not brought forward to policymakers as a source for accessing information and/or perceived as helpful (e.g., given the potentially limited applicability of the evidence, and the format of findings from systematic reviews) [37].

In Lebanon, our documentary analysis revealed that there were multiple efforts to support the use of research evidence in the policy-development process (e.g., systematic reviews syntheses, briefing note and policy dialogue), with none of our participants referencing such efforts in our interviews for two possible reasons. First, this could be a result of our informants’ recollection of activities that occurred in 2014, during interviews conducted in 2018. Second, a case study has shown that although the use of knowledge translation strategies in Lebanon helps to generate evidence-informed policymaking, there is still a need to better link those knowledge translation strategies to specific policy-development processes [38]. For example, an evaluation component should be integrated into the knowledge translation strategy from the start to allow for easier identification of whether and how research evidence was used to inform the policy-development process [38]. This contributes to our understanding on the need for further evaluations that measure the impact of explicit strategies to support evidence-informed policymaking [18, 19].

### Findings in relation to other studies

Our finding show that Lebanon’s and Ontario’s governments shared similar challenges in the perceived scarcity of available research evidence to inform the policy development for Syrian refugees aligns with previous studies identifying that there are perceived gaps in the research evidence to inform policy development about humanitarian crises [13, 16, 39–43]. This study also aligns with other studies that focused on information other than research evidence greatly influencing the policy-development process [16]. In understanding the factors that influence the policy-development process, this study aligns with other studies that suggest how the lack of veto points can support the policy-development process [22, 44–49], policy networks can inform the policy-development process [24, 50–52], values about ‘what ought to be’ can underpin the policy-development process [16, 18], and external factors can be a catalyst of action [53, 54].

### Strengths and limitations

There are four strengths to this study. First, this is the first study to address the knowledge gap in our understanding of the policy-development process for Syrian refugees on two health systems – those of Lebanon and Ontario – operating in very different contexts. Second, the embedded case study design allowed for cross-case analysis thereby providing an in-depth analysis of the factors that influence the policy-development process for Syrian refugees in Lebanon and Ontario and the role of research evidence in the process. Third, we interviewed diverse types of decision-makers encompassing various organizational affiliations to arrive at a comprehensive story of how the policy-development process for Syrian refugees unfolded. Finally, the use of an existing theoretical framework, the only one that provides a comprehensive inventory of the full range of likely political factors, was used to explain the policy-development process for Syrian refugees in Lebanon and Ontario.

One significant challenge to this study related to recruitment of key informants from Ontario’s MOHLTC. Initially there was interest by decision-makers at the MOHLTC to participate in our study with the caveat of waiting until the newly elected government took office. However, after the change of government, we were informed that our request for interviews was declined. We took the following step to address this challenge: we interviewed other key informants who were identified through documentary analysis and by the MOHLTC as actors who were directly involved in the policy-development process.

Another limitation to this study is that the retrieval of media articles in Lebanon was challenging as many of the articles were archived and difficult to access. We addressed this limitation by soliciting the help of a specialized communication officer, who provided us with newspapers articles retrieved from a press tracing exercise conducted on Syrian refugees.

### Implications for policy and practice

The results of our study carry with them some implications. First, this study suggests that other types of information — if not always research evidence — can play an instrumental role in answering specific and direct questions in policy development (e.g., tacit and experiential knowledge used to learn about how best to provide linguistic and culturally appropriate services for Syrian refugees), however, when it comes to a large-scale decision (i.e., addressing health needs of Syrian refugees) other factors are more salient such as the lack of institutional constraints such as veto points. Second, given policymakers’ perception of the scarcity of available research evidence to inform policy development, policymakers not utilizing available evidence websites, and policymakers’ reliance on key stakeholders to share their knowledge and expertise reaffirms the importance for networks to be in place to coordinate and share quality and timely evidence with all stakeholders. For example, EVIPNet is a network established by the World Health Organization to promote the systematic use of data and research evidence in health policymaking by providing key outputs, such as national clearing houses, that aim at providing an opportunity for sharing evidence among all stakeholders [55]. EVIPNet is just one example of the different networks that exist to help policymakers better use research evidence.

### Future research

Our methodology of using an existing theoretical framework, the diversity in our types of decision-makers and organizational affiliation, and key findings on supporting the use of evidence in policy development can be used by researchers studying the policy-development process for Syrian refugees in other host communities (e.g., Turkey, Jordan) and to design and evaluate an intervention to support evidence use in these communities.

## Conclusions

This study suggests that use of research evidence in the policy-development process for Syrian refugees was subordinate to key political factors, resulting in limited influence of research evidence in the development of both the Lebanese and Ontarian policy. This study highlights the need for interested and committed policymakers who value the role of research evidence in informing policymaking.

## Supplementary information

**Additional file 1.** Appendix 1. Data collection and sampling for media, published literature and policy documents.

**Additional file 2.** Appendix 2. Interview guide.

## Data Availability

All data generated or analysed during this study are included in this published article [and its supplementary information files].

## Abbreviations

CRC: Canadian Red Cross
HRS: Health response strategy
K2P: Knowledge to policy
LCRP: Lebanon Crisis Response Plan
LHINs: Local Health Integration Networks
MOHLTC: Ministry of Health and Long-Term Care
MoPH: Ministry of Public Health
NCD: Non-communicable disease
NHSC: National Health Steering Committee
VASyR: Vulnerability Assessment of Syrian Refugees
