# Supplementary material for: The governmental health policy-development process for Syrian refugees: an embedded qualitative case studies in Lebanon and Ontario
Source: Confl Health. 2019 Oct 21;13:48. doi: 10.1186/s13031-019-0231-z (PMC6805559; doi:10.1186/s13031-019-0231-z)
Supplement: Supplementary file 1 — Additional file 1. Appendix 1. Data collection and sampling for media, published literature and policy documents. [file 13031_2019_231_MOESM1_ESM.docx]

**Additional file 1**

# Appendix 1. Data collection and sampling for media, published literature and policy documents

| **Policy** | **Data source** | **Search terms and date of search** | **Included** |
| --- | --- | --- | --- |
| Lebanon’s Health Response Strategy | Published literature-  CINAHL  EMBASE  MEDLINE (PubMed)  SSCI  Web of Science | Terms: “Health polic*” AND “Leban*” AND (“Syria*” OR “refugee*”)  Date: October 15, 2018 | 6 (of 120 studies retrieved)  ([27](#_ENREF_27), [28](#_ENREF_28), [32](#_ENREF_32), [44](#_ENREF_44), [51](#_ENREF_51), [52](#_ENREF_52)) |
|  | Gray literature (e.g., policy documents) | Identified through:   1. Key informant interviews 2. Hand searches of reference lists 3. Google searches | 16 policy documents |
|  | Debates of the Lebanese parliament, laws, ministerial decisions, decrees | Terms: “Syria*” AND *refugee*  Date: November 2, 2018 | 13 (of 6214 references about Syrian refugees in retrieved sessions) |
|  | Newspapers articles retrieved from press tracing exercise | Terms: “refugee” AND “Leban*”  Date: October 16, 2018 | 440 (of 770 individual articles retrieved) |

| Phase 2 Ontario Health System Action Plan: Syrian Refugees | Published literature-  CINAHL  EMBASE  MEDLINE (PubMed)  SSCI  Web of Science | Terms: “Health polic*” AND “Canada*” AND (“Syria*” OR “refugee*”)  Date: June 28, 2018 | 3 (of 363 studies retrieved)  ([29](#_ENREF_29), [30](#_ENREF_30), [50](#_ENREF_50)) |
| --- | --- | --- | --- |
|  | Gray literature (e.g., policy documents) | Identified through:   1. Key informant interviews 2. Hand searches of reference lists 3. Google searches | 8 policy documents |
|  | Hansard: debates of the Legislative Assembly of Ontario | Terms: syria*refugee*  Date: June 28, 2018 | 6 (of 44 references about Syrian refugees in retrieved sessions) |
|  | Newspapers articles –  Lexis Nexis database | Terms: "Syria*" AND "Canad*" AND "health*"  Date: June 28, 2018 | 5 (of 1043 individual articles retrieved) |
