# Supplementary material for: The governmental health policy-development process for Syrian refugees: an embedded qualitative case studies in Lebanon and Ontario
Source: Confl Health. 2019 Oct 21;13:48. doi: 10.1186/s13031-019-0231-z (PMC6805559; doi:10.1186/s13031-019-0231-z)
Supplement: Supplementary file 2 — Additional file 2. Appendix 2. Interview guide. [file 13031_2019_231_MOESM2_ESM.docx]

**Additional file 2**

# Appendix 2. Interview guide

**Introduction**

Hello, my name is X and I want to thank you for taking your time to meet with me today. My study examines the role of research evidence in the health policy-development stage process for [insert policy].

- Denotes probes/prompts

**A. General Questions:**

1. Do you have any questions for me before proceeding with the interview?
2. Which of the following best describes your role in relation to the development of health policies for Syrian refugees:

- policy maker
- manager
- researcher
- healthcare provider
- Syrian refugee

1. What type of organization you are working in:

- government entity (e.g., Ministry of Health, etc.)
- research unit housed in an academic institution, government entity, intergovernmental agency (e.g., UN agencies), NGO
- service provider (e.g., health clinics)

1. To what extent did you get involved in the policy-development/formulation of health polices for Syrian refugees (i.e., Lebanon’s MoPH 2016 Health Response Strategy and Ontario’s2016 Phase 2: Health System Action Plan)?
2. Could you please describe your understanding of the policy-development process in the Lebanese MoPH HRS 2016/Ontario’s Action Plan?

- The main aims of the developed health policies and the problems they were designed to address
- Is there anything about the rules of how these processes worked that might have generally influenced the developed policies?
- Rules put in place from past policies, the openness of the policy-development process to the public, the time-pressured nature of the policy-development process, and the nature of approval required for the policy played a factor in how the policies were developed
- Different stakeholder’s (i.e., Syrian refugees, government, organization) interests played any role in the development of the health polices
- Other sources of ideas
- External factors like an election of a new government influenced how the policies were developed

B. Questions specific **to whether** research evidence was used in the policy-development process of the Lebanese MoPH HRS 2016/Ontario’s Action Plan?

1. Given our discussion earlier, to what extent was research evidence used to develop the Lebanese MoPH HRS 2016/Ontario’s Action Plan?
2. Can you describe to me what type of research evidence you used to inform the Lebanese MoPH HRS 2016/Ontario’s Action Plan?
   - Empirical (e.g., observational studies, surveys and case studies) and conceptual papers (e.g., theoretical papers)
   - Primary or single studies research and secondary research (e.g., systematic reviews and other forms of evidence synthesis)
   - Indexed bibliographic databases or in what is called the grey literature
3. Can you identify if other types of information were used in some of the stages of the policy-development process?
   - Tacit knowledge or ordinary knowledge
   - Stakeholder’s opinions

C. Questions specific to **under what** **conditions** research evidence was used in the policy-development process of the Lebanese MoPH HRS 2016/Ontario’s Action Plan?

1. Is there anything about the rules of how these processes worked that might have influenced the developed policies?

- Rules put in place from past policies, the openness of the policy-development process to the public, the time-pressured nature of the policy-development process, and the nature of approval required for the policy played a factor in how the policies were developed
- Different stakeholder’s (i.e., Syrian refugees, government, organization) interests played any role in the development of the health polices
- Other sources of ideas?
- External factors like an election of a new government influenced how the policies were developed

1. Would you consider the factors you just discussed to have influenced the policy-development stages in a minor or major way?

D. Questions specific to **how** research evidence was used in the policy-development process of the Lebanese MoPH HRS 2016/Ontario’s Action Plan?

1. Can you describe how you used that research evidence?
   - Instrumental: used research evidence in specific and direct ways to solve a specific problem
   - Conceptual: used an overview of reviews of humanitarian-aid interventions to help identify areas where there is a need to give greater or lesser attention
   - Symbolic: used evidence to justify decisions already taken in relation to the developed policies
   - Evidence was used to learn about the benefits, harms, local costs, adaptations and stakeholder’s views and experiences of different options
2. Can you describe how you accessed the research evidence? For example:
   - Reading original research
   - Reading reports produced by policy advisors or interest groups
   - Interacting with researchers or involving researchers in a working group
   - Interacting with peers or stakeholders or involving peers or stakeholders in a working group
   - Attending hearings about the health needs of Syrian refugees

Is there anything else that you could tell me to help me understand the policy-development process in detail?

**Closing remarks:**

- Are there documents (like memos, communications, minutes, etc.) that may help us in identifying the approach to research use employed by your organization for the management of health needs among Syrian refugees?
- Finally, do you know somebody whom do you think may give an important insight to the policy-development process?
- We will be analyzing the information you and others have given. We be sharing the results of the study with you at a later date. In the meantime, thank you for your time.
